# Germination-Driven Modulation of Phenolic Compounds in Sorghum: Bioactivity and Gene Expression

**DOI:** 10.3390/foods15061022

**Published:** 2026-03-14

**Authors:** Raúl Ezerinho Salato, Hugo José Martins Carvalho, Janaina de Oliveira Melo, Marcio Schmiele

**Affiliations:** 1 Laboratório Integrado de Cereais e Lipídeos (LICEL), Instituto de Ciência e Tecnologia (ICT), Universidade Federal dos Vales do Jequitinhonha e Mucuri (UFVJM), Diamantina 39100-000, MG, Brazil; 2 Departamento de Ciências Alimentares, Faculdade de Ciências Agrárias, Universidade Zambeze (UniZambeze), Ulónguè, Tete 0713-02, Mozambique; 3 Departamento de Ciência de Alimentos e Nutrição, Universidade Estadual de Campinas (UNICAMP), Campinas 13083-862, SP, Brazil; 4 Departamento de Ciências Básicas, Universidade Federal dos Vales do Jequitinhonha e Mucuri (UFVJM), Diamantina 39100-000, MG, Brazil; janaina.melo@ufvjm.edu.br; 5 Instituto de Ciências Biológicas, Universidade Federal de Minas Gerais (UFMG), Belo Horizonte 31270-901, MG, Brazil

**Keywords:** antioxidant capacity, bioactive compounds, emerging technologies, phenylpropanoid pathway, secondary metabolism, seed metabolic, sprouting

## Abstract

Controlled germination has emerged as an effective and sustainable bioprocess to enhance the nutritional and functional quality of cereal grains, including sorghum, a climate-resilient crop widely cultivated in tropical and semi-arid regions. Germination triggers coordinated physiological and molecular responses that activate secondary metabolism, particularly the phenylpropanoid pathway, resulting in qualitative and quantitative changes in phenolic compounds. This review summarizes current evidence on germination-driven modulation of sorghum phenolics, with emphasis on bioactivity and genetic regulation. Germination promotes the transcriptional activation of key biosynthetic genes, including those encoding phenylalanine ammonia-lyase and flavonoid pathway enzymes, while regulatory genes associated with condensed tannin biosynthesis, such as *Tannin1* (*Tan*1) and *Tannin2* (*Tan*2), may undergo functional modulation during sprouting, contributing to reduced antinutritional tannin levels. Gene expression and metabolic outcomes are strongly influenced by environmental factors such as soaking duration, temperature, oxygen availability, and elicitation conditions. The resulting phenolic profile exhibits enhanced antioxidant capacity and health-promoting potential. In sorghum-based systems, germination represents a promising strategy to increase bioactive density, reduce antinutritional constraints, and add value to grains. Improved understanding of gene–metabolite interactions during germination may support targeted breeding and the development of functional foods with improved nutritional performance.

## 1. Introduction

Cereals and their derived products are recognized as the primary energy base of the human diet, supplying complex carbohydrates, dietary fiber, and plant-based proteins. Historically, these grains have sustained most global populations, particularly in developing regions, where they frequently represent the main daily caloric source. Beyond their nutritional role, cereals are strategically positioned within modern agri-food systems, contributing to food security and sovereignty, strengthening family-based and subsistence agriculture, and supporting regional socioeconomic development [1,2,3].

In recent decades, the use of cereals in human nutrition has undergone a substantial transformation, driven by climate change, shifts in dietary patterns, scientific and technological progress, and increasing concerns related to human health and the sustainability of food systems [4]. Hence, processing strategies such as biofortification, controlled germination, fermentation, extrusion, and related technologies have been extensively investigated as means to enhance nutrient bioavailability, reduce antinutritional factors, and improve the nutritional and technological functionality of cereal grains [5]. Germination has been widely recognized as an effective bioprocessing strategy capable of improving the nutritional and functional quality of plant-based foods when compared with conventional post-harvest treatments. This physiological process induces intense metabolic activity within the seed, promoting the activation of endogenous enzymatic systems and stimulating the biosynthesis of secondary metabolites, particularly phenolic compounds associated with the phenylpropanoid pathway. As a result, germination has been associated with increased antioxidant capacity and a reduction in antinutritional factors. During germination, the activity of hydrolytic enzymes such as cellulases, hemicellulases, and pectinases promotes the partial degradation of cell wall components and structural rearrangements within the grain matrix. These enzymatic modifications facilitate the disruption of interactions between phenolic compounds and macromolecules of the cell wall, including polysaccharides and structural proteins. Consequently, phenolic compounds that were previously bound or entrapped within the cellular matrix become more extractable and bioaccessible, which may enhance their antioxidant potential and biological functionality [3,5].

Among the most widely produced cereals worldwide, maize, rice, wheat, and barley rank first, followed by sorghum, which occupies the fifth position and exhibits diverse applications in human and animal nutrition, as well as in environmental remediation, including phytoremediation of contaminated soils [6]. Sorghum (*Sorghum bicolor* L. Moench), a cereal of African origin, is extensively cultivated in tropical and subtropical regions and is characterized by high agronomic performance and remarkable adaptability to adverse conditions, including water scarcity and intense biotic stress. Additionally, lower production costs compared with major cereals such as maize have increased its suitability for low-input and family-based agricultural systems [7,8].

From a nutritional and functional standpoint, sorghum has received growing international attention due to its natural absence of gluten-forming proteins, its high dietary fiber content (approximately 10%, including resistant starch), and its broad diversity of bioactive compounds capable of modulating metabolic processes and promoting health benefits [6]. Although sorghum represents a major source of energy and nutrients for populations in several African and Asian countries [9], its production in many regions remains predominantly directed toward animal feed. Under this perspective, sorghum presents considerable potential for expanded use as a functional and health-promoting food ingredient, integrating favorable agronomic traits, advances in genetic improvement, and increasing consumer demand for sustainable and nutritious foods consistent with circular economy principles [10].

Accordingly, this review was conducted to provide a comprehensive synthesis of current knowledge on sorghum, with emphasis on grain morphology, proximate composition, phenolic compounds, bioactive properties, antinutritional factors, and gene expression mechanisms involved in the synthesis and bioconversion of phenolic compounds.

## 2. Methodology

This literature review was conducted as systematic literature review: a methodological approach that enables the systematic and rigorous synthesis of existing knowledge through a structured analytical process. This study was conducted following the recommendations of the PRISMA (Preferred Reporting Items for Systematic Reviews and Meta-Analyses) guidelines. Scientific articles addressing phenolic compounds, bioactivity, and gene expression in sorghum, particularly in relation to germination processes. The literature search was performed in the following electronic databases: ScienceDirect, Scopus, SciELO, PubMed (MEDLINE), and the CAPES/MEC Journal Portal. Only peer-reviewed articles published in English were considered. The selection was limited to studies published within the last 15 years (2010–2025) and the search strategy used specific descriptors: (Sorghum OR “*Sorghum bicolor*”) AND (germination OR sprouting OR soaking) AND (“phenolic compounds” OR polyphenol OR flavonoid OR “phenolic acids” OR “3-deoxyanthocyanidin” OR tannin) AND (“gene expression” OR “transcriptional activation” OR ”genetic regulation” OR “metabolic pathway” OR “phenylpropanoid pathway” OR “phenylalanine ammonia-lyase” OR “PAL”) AND (“antioxidant capacity” OR “health-promoting” OR “bioactivity”). 

Studies were screened based on their titles and abstracts to assess relevance to the objectives of the review. Subsequently, full-text articles were evaluated for eligibility. Duplicate records identified across the databases were removed prior to screening. Articles that did not address the effects of germination or related processes on phenolic composition, bioactivity, or gene expression in sorghum were excluded.

Articles that did not align with the objectives of this review or that were identified as duplicates across databases were excluded. This selection strategy ensured a comprehensive, focused, and up-to-date synthesis of scientific evidence regarding the effects of germination and related processes on phenolic composition and gene expression in sorghum. Artificial intelligence-assisted language editing was performed using ChatGPT, version GPT-5.0 Plus (OpenAI, San Francisco, CA, USA) to support translation and grammatical refinement from Portuguese into English. Scientific figures were created using BioRender, version Premium (BioRender.com, Toronto, ON, Canada).

## 3. Characterization of the Sorghum Grain

**Figure 1:**
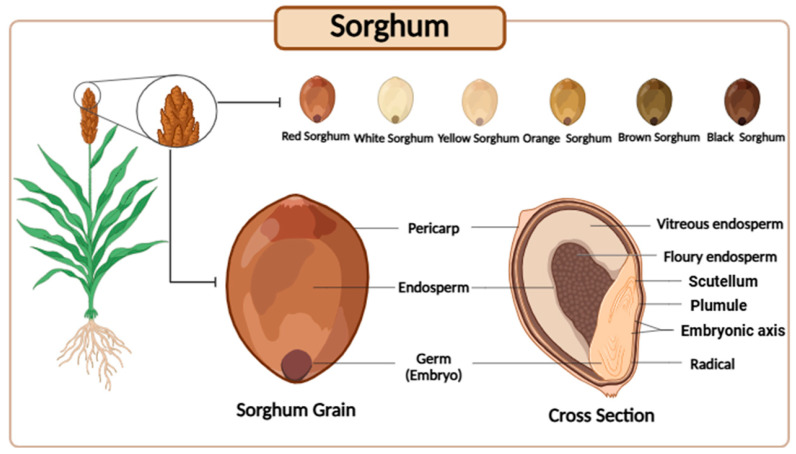
Morphological structure of sorghum grains from different varieties.

The sorghum grain is anatomically composed of three distinct fractions: the pericarp, endosperm, and germ (Figure 1). The pericarp is enriched in non-starch polysaccharides, minerals, and a diverse range of phenolic compounds, including phenolic acids, flavonoids, and condensed tannins. The endosperm consists predominantly of starch and storage proteins, with minor amounts of vitamins, whereas the germ fraction is primarily composed of lipids and soluble sugars, in addition to vitamins and minerals [7,10].

Among these fractions, the pericarp has received particular attention due to its high phytochemical density, especially of bioactive phenolic compounds, whose concentrations may reach levels up to six-fold higher than those observed in the whole grain. This feature identifies the pericarp as a strategic target for valorization in the development of functional food ingredients. Pericarp coloration varies from white and yellow to red and brown; although color alone is not a definitive indicator of phenolic content, higher polyphenol concentrations are generally associated with darker, particularly brown, grain phenotypes [7,11].

## 4. Nutritional Composition

Knowledge of the nutritional composition and constituent profile of foods, whether raw or processed, is essential for ensuring adequate human nutrition and food security. Sorghum exhibits a nutritional profile comparable to that of other major cereals, with starch as its predominant component, typically ranging from approximately 50 to 72.5 g·100 g^−1^. This starch fraction is largely classified as slowly digestible, a characteristic associated with favorable postprandial metabolic responses.

Dietary fiber content in sorghum generally ranges from 6 to 15 g·100 g^−1^, with hemicellulosic fractions predominating and accounting for approximately 75% of the insoluble dietary fiber [10,12]. Protein content typically ranges from 7 to 15 g·100 g^−1^, with nearly 79% represented by prolamins, mainly α-, β-, and γ-kafirins. Although the total lipid content of sorghum is relatively low (<3 g·100 g^−1^), its lipid fraction is characterized by a high proportion of unsaturated fatty acids, including oleic, linoleic, and linolenic acids, along with notable levels of palmitic and stearic acids [12].

Sorghum also provides essential minerals such as potassium, sulfur, magnesium, phosphorus, zinc, and copper, as well as B-complex vitamins, including thiamine, riboflavin, niacin, pyridoxine, and folate. These micronutrients are predominantly located in the outer grain layers and may be substantially reduced during refining processes. In addition, the germ fraction constitutes an important source of vitamin E, while yellow sorghum varieties contain provitamin A compounds, particularly carotenoids [10,12].

## 5. Phenolic Compounds and Functional Properties

**Figure 2:**
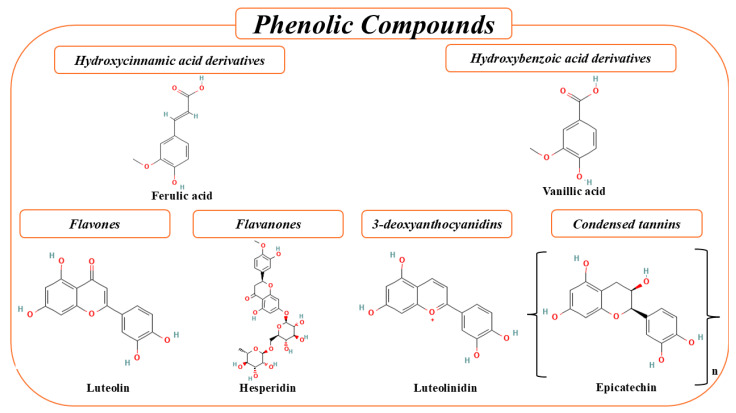
Main classes of bioactive compounds present in sorghum.

In sorghum, the major bioactive constituents are predominantly phenolic compounds. The principal phenolic classes include phenolic acids, flavonoids, and condensed tannins, which are biosynthesized via the phenylpropanoid pathway, as illustrated in Figure 2 and summarized in Table 1. The concentration and qualitative composition of these compounds in sorghum grains are modulated by multiple factors, particularly genotype and edaphoclimatic conditions [2]. Sorghum phenolics have been extensively investigated due to their broad spectrum of biological activities, including antioxidant, antidiabetic, anticarcinogenic, anti-inflammatory, antimicrobial, antihypertensive, anti-aging, and neuroprotective effects, underscoring their relevance in health promotion and disease prevention [13,14].

The polyphenolic profile of sorghum is characterized by substantial variability among cultivars (Table 1), being strongly associated with grain coloration and additionally influenced by the analytical methods employed and the phenolic fraction evaluated (soluble or bound). In general, sorghum varieties with darker pigmentation, such as red, brown, and black, are characterized by higher concentrations of total phenolics, flavonoids, and proanthocyanidins, resulting in a more complex and concentrated bioactive profile and greater antioxidant potential compared with lighter-colored genotypes, including white, yellow, pearled, and orange varieties. Sorghum with a red pericarp is recognized as an important source of 3-deoxyanthocyanidins. In general, the intensity of pericarp pigmentation is positively associated with the concentration of these compounds, such that grains with darker pericarp colors (particularly brown and black) tend to present higher levels of 3-deoxyanthocyanidins and condensed tannins [18,19,20].

Regarding individual phenolic composition, hydroxycinnamic acid derivatives (particularly caffeic, *p*-coumaric, and ferulic acids) are the most consistently reported across sorghum varieties. These compounds are predominantly associated with the pericarp fraction of pigmented sorghum grains and contribute substantially to antioxidant activity and oxidative stability in sorghum-derived products. Comparable patterns are observed for hydroxybenzoic acids, including protocatechuic and gallic acids, further underscoring the chemical diversity of the sorghum phenolic profile [6,20].

The predominance of 3-deoxyanthocyanidins, such as luteolinidin and apigeninidin, is a defining characteristic of sorghum varieties exhibiting red, brown, and black pigmentation. These pigments are largely unique to sorghum and play a central role in determining grain coloration. Compared with conventional anthocyanins, 3-deoxyanthocyanidins exhibit relatively greater chemical stability, particularly under mildly acidic to neutral conditions, due to the absence of the hydroxyl group at the C-3 position, which reduces susceptibility to hydration and structural degradation. This enhanced stability increases their potential applicability as natural colorants and bioactive compounds in food systems. Additional flavonoid subclasses, including flavones (luteolin and apigenin), flavanones (naringenin and its glycosylated derivatives), and flavonols (quercetin and rutin), further complement the polyphenolic profile, albeit generally at lower concentrations [21,22].

### 5.1. Phenolic Acids

Phenolic acids constitute the most abundant class of phenolic compounds in sorghum grains, with reported total concentrations ranging from 445 to 2850 µg·g^−1^ [14]. Based on structural characteristics, these compounds are classified into hydroxybenzoic acids, derived from benzoic acid, and hydroxycinnamic acids, derived from cinnamic acid [23]. Hydroxybenzoic acids exhibit a C6–C1 backbone and include gallic, salicylic, ellagic, protocatechuic, syringic, and vanillic acids, which differ according to the degree and position of substitutions on the aromatic ring [2].

In contrast, hydroxycinnamic acids possess a C6–C3 structure and include *p*-coumaric, cinnamic, caffeic, and ferulic acids. Structural variability within this group arises from differences in side-chain length and the number and positioning of hydroxyl substituents on the aromatic ring [1]. Compared with hydroxybenzoic acids, hydroxycinnamic acids generally exhibit higher antioxidant activity, which is attributed to the presence of conjugated double bonds in the propanoid side chain, facilitating electron delocalization and enhanced radical-scavenging capacity [23].

In sorghum grains, phenolic acids are predominantly localized in the pericarp, with lower concentrations detected in the endosperm, and occur in both free and bound forms. Free phenolic acids are mainly present as esterified conjugates linked to mono- and oligosaccharides or glycerol, whereas bound phenolic acids are covalently associated with cell wall components, forming an integral part of the grain structural matrix. Notably, approximately 70–95% of phenolic acids in sorghum are present in the bound form [7,24].

Despite their abundance, bound phenolic acids exhibit limited bioavailability due to extensive covalent cross-linking that confers resistance to enzymatic hydrolysis. Furthermore, phenolic acid distribution has been associated with kernel hardness, which influences extractability and nutritional functionality [25]. Phenolic acids exert antioxidant activity primarily through hydrogen atom or electron donation, thereby delaying or inhibiting biomolecular oxidation. This activity is strongly structure-dependent and generally increases with the number of hydroxyl groups present in the molecule [26].

### 5.2. Flavonoids

Flavonoids are a diverse group of phytochemicals characterized by a C6–C3–C6 backbone, in which two aromatic rings (A and B) are linked by a central heterocyclic ring (C). These compounds are typically localized within vacuoles or associated with cell wall structures. In sorghum grains, flavonoids constitute one of the largest and most diverse classes of phenolic compounds and are predominantly concentrated in the pericarp, where their accumulation is closely associated with grain pigmentation.

As naturally occurring food constituents, flavonoids have been widely associated with a variety of beneficial biological activities and may contribute to positive health-related effects. Consequently, these compounds have attracted considerable interest in their applications in food systems. Within sorghum grains, the predominant flavonoid subclasses include 3-deoxyanthocyanidins, flavones, and flavanones, which largely define the distinctive polyphenolic profile and bioactivity of this cereal [14,22].

#### 5.2.1. Flavones

Flavones are yellow-pigmented flavonoids that contribute to human nutrition due to their ability to neutralize free radicals and protect cells against oxidative stress. In sorghum grains, however, flavone concentrations are relatively low compared with other flavonoid subclasses, typically ranging from 20 to 390 µg·g^−1^ [14,27].

In cereals, flavones predominantly occur in glycosylated forms, with O-glycosides representing the most frequently identified structures in sorghum grains. These compounds tend to exhibit relatively high bioavailability due to their susceptibility to hydrolysis under gastric acidic conditions, which may result in appreciable bioactivity even at low concentrations [27]. Sorghum varieties with red and yellow pericarps have been reported to contain higher flavone levels compared with other grain color types [28].

#### 5.2.2. Flavanones

Flavanones are widely distributed among edible plants and are recognized as key intermediates in flavonoid biosynthesis; however, they are rarely detected in cereal grains [7]. Sorghum represents a notable exception, with reported flavanone concentrations ranging from 0 to 2000 µg·g^−1^. The lowest levels are generally observed in white sorghum varieties, whereas the highest concentrations are typically associated with yellow-colored genotypes [28].

Flavanones exhibit multiple health-promoting effects, including antioxidant, anti-inflammatory, and antitumor activities, and have also been associated with beneficial modulation of gut microbiota and intestinal health. Similar to flavones, flavanones in sorghum are mainly present as *O*-glycosides, which are readily hydrolyzed under low pH conditions, resulting in high bioavailability [29,30].

#### 5.2.3. 3-Deoxyanthocyanidins

The 3-deoxyanthocyanidins constitute a unique subclass of anthocyanins found almost exclusively in sorghum grains. This group includes luteolinidin, apigeninidin, 5-methoxyluteolinidin, and 7-methoxyapigeninidin, which are derived from the flavanone biosynthetic pathway. Structurally, these compounds differ from conventional anthocyanins by the absence of a hydroxyl group at the C-3 position, a feature that confers distinctive chemical stability and functional properties [7].

3-Deoxyanthocyanidins represent the most abundant flavonoid subclass in sorghum, with total concentrations ranging from 200 to 4500 µg·g^−1^. In specific genotypes, such as BRS-305, they may account for up to 80% of the total flavonoid content of the grain [14]. Varieties with red, black, and purple pericarps exhibit particularly high concentrations (1790–6120 µg·g^−1^), and these compounds are primarily responsible for intense grain pigmentation. Accumulation occurs mainly in the pericarp and testa, whereas white sorghum varieties exhibit low levels or complete absence, resulting in lighter grain coloration [31].

Beyond their role in pigmentation, 3-deoxyanthocyanidins exhibit strong antioxidant activity and demonstrate antimicrobial, anti-inflammatory, and anticarcinogenic properties [14]. Furthermore, owing to their water solubility and superior color stability compared with conventional anthocyanins, these compounds show significant potential for application as natural food colorants and functional ingredients [22].

## 6. Antinutrients

The consumption of sorghum in human diets remains limited in certain regions due to the presence of compounds considered antinutritional, which may impair nutrient, proteins and starch digestibility. The principal antinutritional factors in sorghum grains are phytic acid, proteins, especially kafirins, and tannins, which form complexes with starch, proteins, and minerals. Interactions between polyphenols and major grain macromolecules, particularly starch and proteins, can lead to the formation of complexes such as polyphenol–starch, starch–kafirin, and tannin–protein. These interactions may enhance resistance to enzymatic hydrolysis, thereby reducing nutrient bioavailability and increasing the proportion of resistant starch [32].

### 6.1. Phytic Acid

Phytic acid content in sorghum grains may reach up to 1% and represents the primary storage form of phosphorus, being predominantly localized in the pericarp and germ. Owing to its strong chelating capacity, phytic acid reduces the bioavailability of essential minerals such as iron, zinc, calcium, and magnesium, and forms insoluble complexes with proteins, digestive enzymes, and lipids, resulting in impaired nutrient digestibility and absorption. Consequently, phytic acid is widely recognized as an antinutritional factor in sorghum-based foods [33,34,35]. Nevertheless, beneficial physiological effects have also been reported, including antioxidant activity and potential anticancer properties in vitro. Phytic acid concentration in whole sorghum grains is influenced by genotype, edaphoclimatic conditions, agricultural practices, and processing intensity [36,37].

### 6.2. Tannins

Tannins are non-flavonoid phenolic compounds widely distributed in plants, occurring as low-molecular-weight monomers or oligomers. In sorghum, tannins are predominantly present in condensed forms with high molecular weight, mainly composed of flavan-3-ols and flavan-3,4-diols, and characterized by a high degree of polymerization [31,38].

Based on tannin concentration and genetic background, sorghum grains are commonly classified into three types: Type I sorghum, carrying recessive B1 and B2 genes and exhibiting low or negligible tannin levels (0–1.8 mg CE·g^−1^); Type II sorghum, characterized by pigmented pericarps with dominant B1 and B2 genes and a recessive S gene, showing moderate tannin levels (6.4–15.5 mg CE·g^−1^); and Type III sorghum, possessing dominant B1, B2, and S genes and exhibiting high tannin contents (11–50.2 mg CE·g^−1^) [38,39].

Elevated tannin concentrations are associated with bitterness and astringency in sorghum grains. In addition to sensory effects, tannins bind to proteins and minerals, impair enzyme activity, reduce hydrolysis rates of macronutrients (starch, proteins, and lipids), and consequently decrease the nutritional and caloric value of the cereal [38,40]. However, similar to phytic acid, tannins also exhibit health-promoting properties, particularly antioxidant activity [41].

Tannins interact with dietary starches and proteins, affecting nutrient digestibility and bioavailability, and may chelate metal ions such as iron and copper, which catalyze oxidative reactions. By limiting the availability of these metals, oxidative damage in biological systems may be mitigated. Conversely, interactions with calcium and magnesium salts may reduce the bioavailability of these minerals [42,43].

Due to their complex chemical structure, characterized by multiple aromatic rings and hydroxyl groups, tannins are not degraded during gastric transit in humans. Instead, they are metabolized by the colonic microbiota, resulting in the formation of phenolic acids that may subsequently be absorbed. Therefore, tannins in sorghum exhibit an ambivalent nature, acting simultaneously as bioactive compounds and antinutritional factors. Their overall impact on grain quality is determined by concentration, processing strategies applied, and the dietary context in which sorghum is consumed [44,45,46].

## 7. Importance of Bioactive Compounds in Health Promotion

Phenolic compounds derived from cereals are recognized as important contributors to human health following digestion within biological systems. Protective effects may be exerted upon reaching the gastrointestinal tract, where interactions with the gut microbiota promote their biotransformation into bioactive metabolites. Through these interactions, phenolic compounds contribute to intestinal homeostasis and may confer systemic health benefits [47,48].

Conversely, antioxidant and anti-inflammatory biological functions of bioactive compounds require the absorption of phenolic compounds or their metabolites into the circulatory system, allowing distribution to specific target cells and tissues [7]. Accordingly, bioactive compounds may exert health-promoting effects through both direct and indirect mechanisms involving complex biochemical interactions. These effects are not restricted to localized sites but may act systemically, contributing to the modulation of physiological processes associated with disease prevention and progression [7,47].

The synthesis, release, and bioconversion of phenolic compounds in cereals involve multiple biosynthetic precursors, particularly the amino acids phenylalanine and tyrosine [23]. Integration of metabolomics with genomic and sequencing-based approaches has enabled improved characterization of the structure, function, and health effects of cereal-derived phenolics, providing deeper insight into their genetic regulation [38,49,50].

Several emerging processing strategies have been explored to further enhance the accumulation and bioavailability of phenolic compounds in cereals and pseudocereals, as comprehensively described by Minchán-Velayarce and coauthors [5]. These approaches generally rely on the induction of controlled metabolic stress or structural modifications within the grain matrix, thereby stimulating the biosynthesis or release of phenolic compounds. For instance, fermentation, abiotic elicitation through exposure to salinity, temperature variations, or oxygen limitation has been reported to activate key enzymes of the phenylpropanoid pathway, particularly phenylalanine ammonia-lyase (PAL), leading to increased phenolic synthesis. Similarly, exposure to ultraviolet radiation or controlled light conditions may stimulate the production of flavonoids and other antioxidant metabolites. Physical technologies, such as ultrasound-assisted germination, cold plasma treatment, and pulsed electric fields, have also demonstrated promising results by enhancing cell permeability and facilitating the release of phenolic compounds bound to the cell wall matrix. In addition, enzymatic treatments involving cellulases, hemicellulases, and β-glucosidases can promote the hydrolysis of structural polysaccharides, thereby increasing the extractability and bioaccessibility of phenolic compounds. Collectively, these emerging strategies represent promising complementary approaches to conventional germination processes and may contribute to the development of cereal-based foods with improved nutritional and functional attributes.

Phenolic composition in cereals is strongly influenced by the germination process, which modulates metabolic pathways and bioactive profiles [51].

## 8. Germination as an Emerging Technology

In recent decades, the increasing food demand of a rapidly growing global population has been largely addressed through gains in agricultural productivity; however, yield enhancement alone is increasingly recognized as insufficient to meet nutritional requirements. Consequently, innovation in food processing technologies has been emphasized as a key strategy to extend shelf life, optimize nutrient availability, and improve overall food quality [52]. Simultaneously, the production of healthy diets that align with consumer expectations has been required to rely on accessible and scalable technologies in a global context characterized by resource constraints, demographic transitions, and increasing urbanization [53].

Cereal crops, as staple foods in many regions, have therefore received heightened attention, with sorghum emerging as a particularly relevant crop due to its exceptional adaptability to diverse and challenging agroclimatic conditions when compared with other cereals [7]. Despite these advantages, the nutritional and functional potential of sorghum has historically been underexploited, prompting the development of processing strategies aimed at enhancing its value in response to the growing demand for health-oriented foods [20].

The pursuit of high-quality functional foods has been extensively explored by the food industry, and biotechnological processes such as germination and fermentation have emerged as promising approaches for sorghum processing, as they facilitate the release and bioconversion of phenolic compounds with health-promoting properties [54,55]. Although germination represents one of the oldest food-processing techniques and is widely applied in beverage production, particularly brewing, its renewed relevance is attributed to its classification as a green and sustainable bioprocess. This process relies primarily on water and temperature, minimizing the use of chemical additives [4]. Moreover, germination functions as a natural biofactory capable of inducing molecular-level modifications and may be integrated with emerging physical (e.g., cold plasma, ultrasound, ultraviolet light) and biological (e.g., gibberellic acid, amino acids) technologies to further enhance its effectiveness [5].

## 9. Changes During Germination Process

Cereals currently represent one of the major contributors to the human diet worldwide, driven by growing interest in healthy nutrition. They are widely recognized as safe and reliable sources of energy, macro- and micronutrients, and health-promoting compounds [56]. To overcome nutritional limitations associated with cereals, particularly the presence of antinutritional factors, germination has emerged as an effective and sustainable processing alternative [55].

Germination is a biochemical process characterized by the termination of seed dormancy. During this process, endogenous enzymes are activated and released, playing a central role in the hydrolysis of macromolecules into simpler components, thereby enhancing nutrient digestibility and bioavailability. Simultaneously, germination promotes the synthesis, release, and bioconversion of secondary metabolites with recognized health benefits. Consequently, germination has been widely adopted as a strategy to improve the nutritional quality of foods intended for health-oriented diets [57,58].

The enzymatic activity induced during germination results in profound modifications to the nutritional and functional composition of grains, increasing nutrient availability while reducing antinutritional factors (such as protease inhibitors and phytic acid) through enzymatic hydrolysis. In parallel, the biosynthesis of vitamins and phytonutrients, particularly phenolic compounds, is stimulated [59,60]. These changes are influenced by multiple factors, including seed genotype and germination conditions such as temperature, duration, relative humidity, light exposure, CO_2_ availability, pre-treatment protocols, water availability, and the application of elicitors [5,61]. During the germination process, intense metabolic activity occurs, characterized by physiological, biochemical, and molecular changes that favor seedling establishment. At this stage, secondary metabolism is activated, particularly the biosynthesis of phenolic compounds, and different environmental elicitors can modulate this process by acting as regulatory signals that induce defense mechanisms and cellular adaptation. Among the main elicitors are light, ultraviolet radiation, and biotic stressors [5]. Light is perceived by plant photoreceptors such as phytochromes and cryptochromes. These receptors activate a series of signaling pathways that regulate the expression of genes involved in phenolic metabolism, including key enzymes such as PAL, which is responsible for the initial step of the phenylpropanoid pathway. Another abiotic elicitor is UV-B radiation, which promotes the generation of reactive oxygen species (ROS) and triggers oxidative stress responses. In addition to physical stimuli, biotic elicitors also influence metabolism during germination. The presence of microorganisms or pathogen-derived molecules can activate molecular recognition receptors in the seedling. This process triggers signaling cascades involving regulatory molecules such as salicylic acid, resulting in the activation of genes associated with the production of defense metabolites, including phenolic compounds and phytoalexins [5,60,61].

### 9.1. Influence of Germination on the Phenolic Profile

Phenolic compounds are synthesized within intracellular organelles, primarily the endoplasmic reticulum, and are subsequently transported via vesicular trafficking to vacuoles or incorporated into the cell wall. Soluble phenolics predominantly accumulate in vacuoles, whereas insoluble phenolics are mainly associated with cell wall structures. During germination, the induction of enzymes capable of hydrolyzing cell wall polysaccharides promotes cell wall loosening and increased permeability, facilitating endosperm rupture and enhancing the exposure of nutrients and bioactive compounds to enzymatic action [56,62,63].

The observed increase in extractable phenolic compounds during germination has been attributed to the release of hydrolytic enzymes (lipases, proteases, and carbohydrases) from the aleurone layer into the endosperm, leading to the degradation of complex macromolecular matrices [64]. As a result, both biosynthetic and hydrolytic enzymatic activities dominate the germination process, promoting qualitative and quantitative modifications in phenolic composition [65]. The effects of germination on the phenolic profiles of different sorghum varieties under varying germination conditions are summarized in Table 2.

Seed germination plays a critical role in increasing phenolic content, thereby enhancing nutritional value and associated health benefits. This effect is primarily attributed to endogenous enzyme activation, macromolecular bioconversion, and intensified metabolic activity initiated by water uptake and seed activation. Elevated phenolic levels are further associated with the upregulation of the phenylpropanoid biosynthetic pathway, particularly through the activation of phenylalanine ammonia-lyase (PAL), a key regulatory enzyme in this pathway. Such activation is directly linked to increased expression of genes involved in phenylpropanoid metabolism, resulting in the enhanced biosynthesis of phenolic acids and flavonoids [71,72,73].

However, depending on germination conditions and seed genotype, reductions in total phenolic content may also be observed, primarily due to the solubilization and leaching of soluble phenolics during seed sanitization and soaking steps [68,71]. Germination additionally induces seed defense responses, promoting the biosynthesis of both soluble and bound phenolics via the activation of photosynthetic activity and cellular metabolism as protective mechanisms against abiotic stresses, including photo-oxidation and reactive oxygen species (ROS) generation. As summarized in Table 2, decreases in specific phenolic compounds may result from condensation and polymerization reactions catalyzed by enzymes such as polyphenol oxidases [67,69].

Furthermore, enhanced activity of hydrolytic enzymes, including cellulases, amylases, and polyphenol oxidases, may favor polymerization and structural modification of phenolic compounds, with outcomes dependent on processing conditions such as temperature, duration, humidity, and seed variety [62,66,68]. Germination also activates interconnected secondary metabolic pathways, including the oxidative pentose phosphate pathway, glycolysis, the acetate–malonate pathway, the shikimate pathway, and the phenylpropanoid pathway, collectively contributing to dynamic modulation of phenolic composition [61].

### 9.2. Effect of Germination on Tannins

Grains provide a broad spectrum of nutrients [74]. These constituents negatively affect nutritional quality and functional properties and may increase processing requirements. In sorghum, tannins are regarded as the primary antinutritional factors [72,73].

Tannins are polyphenolic compounds that reduce amino acid availability in cereals through interactions with protein carbonyl groups [75]. Despite these antinutritional effects, tannins have also attracted considerable scientific interest due to their potential beneficial biological properties, including antioxidant activity and possible protective effects associated with the modulation of oxidative stress. During germination, the activation of catabolic enzymes (particularly polyphenol oxidases) facilitates the degradation of polyphenolic compounds, resulting in a progressive reduction in tannin content. In addition, tannins may be partially removed through leaching into soaking water during seed maceration, further contributing to their decrease [76,77].

## 10. Genetic Regulation of Bioactive Compounds in Sorghum During Grain Maturation and Germination

Understanding the biosynthetic pathways of bioactive compounds and their genetic regulation is essential for improving the nutritional and functional quality of foods. In recent years, increasing attention has been directed toward the identification and characterization of genes involved in the biosynthesis of these compounds, as both nutritional composition and bioactive profiles are strongly genotype-dependent [67,78]. Consequently, the mapping of structural and regulatory genes governing bioactive biosynthetic pathways has become a fundamental strategy in cereal breeding programs aimed at developing varieties with enhanced bioactive content and improved bioavailability [38]. These efforts have encompassed major cereal crops, including maize, wheat, rice, and sorghum [79,80], and have been substantially advanced by the application of modern molecular biology tools, particularly genomics [50] and transcriptomics [81].

Among the bioactive compounds most relevant to human health in sorghum are phenolic acids and flavonoids, with particular emphasis on 3-deoxyanthocyanidins and condensed tannins [14,28].

### 10.1. Biosynthesis of Bioactive Compounds During Grain Maturation

In general, pigmented sorghum varieties exhibit higher tannin contents, with greater concentrations commonly reported in brown and red grains [7,41]. Pericarp color is determined primarily by the *R* and *Y* loci. When both genes are dominant, a red-grain phenotype is observed (*R_—_Y_—_*); when both are recessive (*rryy*), grains are white; and when *Y* is dominant in an *rr* background (*rrY_—_*), grains display a yellow phenotype [7]. The *B1* and *B2* loci regulate tannin biosynthesis and also influence grain color together with the *S* (*Spreader*) locus, which controls pigment dispersion in the testa. Genotypes carrying dominant alleles at *B1*, *B2*, and *S* typically exhibit higher tannin levels and are frequently associated with brown-grain phenotypes [7,82].

**Figure 3:**
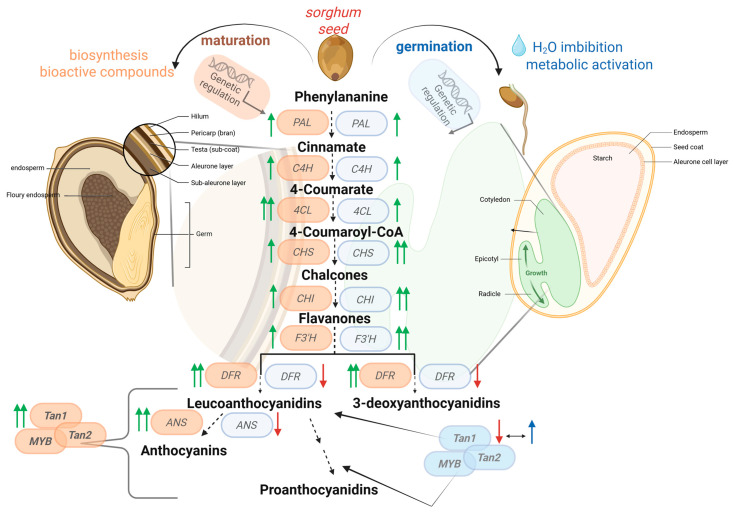
Schematic representation summarizing the phenylpropanoid pathway involved in the biosynthesis of flavonoids and tannins during sorghum grain maturation and germination. On the left side, the grain during the maturation phase is depicted and, on the right side, grain germination is represented. Circles represent genes: brown circles indicate the expected gene expression trend during grain maturation, whereas blue circles indicate the expected trend during germination. Overlapping circles represent proteins that form complexes. Dashed arrows summarize the biosynthetic pathway, in which some intermediate steps may have been omitted for clarity. Green arrows indicate increased expression, red arrows indicate reduced expression, blue arrows indicate stable expression, double arrows indicate strong upregulation (overexpression) and the black double arrows suggest downregulated or stable expression. *PAL*: phenylanaline amonialyase; *C4H*: cinamato-4-hidroxilase; *4CL*: 4-coumarato-CoA ligase; *CHS*: chalcone synthase; *CHI*: chalcone-flavanone isomerase; *F3′H*: flavanone 3′-hydroxylase; *DFR*: dihydroflavonol-4-reductase; *ANS*: anthocyanidin synthase; *Tan1*: *Tannin1*; *Tan2*: *Tannin2*. Genome-wide association studies (GWAS) have enabled the identification of quantitative trait loci (QTLs) associated with sorghum bioactive traits, including antioxidant capacity, total polyphenols, tannins, anthocyanins, and 3-deoxyanthocyanidins [11,77,87,88,89]. However, many QTL, particularly those for antioxidant capacity, phenolics, and anthocyanins, exhibit small effect sizes, underscoring the need for additional studies. Ref. [88] reported significant associations between SNPs near *Tan1* and 13 additional SNPs potentially linked to candidate genes involved in flavonoid biosynthesis. Associative mapping further identified new loci related to polyphenols and revealed association peaks on chromosome 4 co-localized with *Tan1*, along with other genes involved in flavonoid biosynthesis [11,84]. A significant SNP on chromosome 2—homologous to Arabidopsis *TT8*—was also detected, suggesting an association with *Tan2* [11,84]. In a GWAS of 96 sorghum genotypes, two main SNPs associated with total phenolics and luteolinidin were linked to the genes *PDHA1* and *LeETR4* [89]. *PDHA1* participates in the conversion of pyruvate to acetyl-CoA, whereas *LeETR4* belongs to an ethylene receptor subfamily, suggesting a regulatory role in ethylene signaling. *LeETR4* expression was associated with total phenolics and luteolinidin content [89].

Condensed tannins are synthesized in the seed testa of pigmented genotypes [38] during grain maturation through enzymes and proteins localized in the endoplasmic reticulum membranes or cytoplasm. These are encoded by structural and regulatory genes that coordinate a series of biochemical reactions converting phenolic precursors into complex compounds within a specialized branch of the flavonoid pathway (Figure 3). In sorghum, *Tannin1* (*Tan1*) was identified as a key regulatory gene for condensed tannin synthesis [38]. *Tan1* is homologous to the Arabidopsis *TTG1* gene, is located on sorghum chromosome 4, and encodes a WD40 transcriptional regulator that controls tannin biosynthesis [38]. Molecular characterization of *Tan1* revealed three mutations leading to non-functional alleles and a tannin-free phenotype: (i) a G/C single-base deletion in the coding region, (ii) a G→T substitution, and (iii) a 10 bp insertion [38,83]. Several recessive loss-of-function alleles (*tan1–a*, *–b*, *–c*) and newly reported alleles (*tan1–d* and *tan1–e*) contain deletions in the coding region and also confer the tannin-free phenotype [84,85].

Subsequently, *Tannin2* (*Tan2*) was identified on sorghum chromosome 2 as a homolog of Arabidopsis *TT8*. *Tan2* encodes a basic helix–loop–helix (bHLH) protein that interacts with the WD40 protein encoded by *Tan1* and is also homologous to *Rc* (rice) and *IN1* (maize) [84]. Mutations in *Tan2* likewise result in tannin-free phenotypes in sorghum genotypes recessive at this locus [84,85]. In addition to known recessive alleles (*tan2–a*, *–b*, *–c*), a new allele (*tan2–d*) was described involving a C→T transition that introduces a premature stop codon upstream of the bHLH domain. Importantly, tannin-free sorghum can result from homozygous recessive mutations in either *Tan1* or *Tan2* [84,85].

*Tan2* displays epistatic interaction with *Tan1* in controlling tannin biosynthesis. *Tan1* and *Tan2* correspond to the classical *B1* and *B2* loci, respectively, mapped for tannin-present versus tannin-absent phenotypes. Mutations in either gene lead to the absence of condensed tannins [85,86]. Together, the transcription factors encoded by *Tan1* (WD40), *Tan2* (bHLH), and an MYB protein (whose gene has not yet been fully characterized in sorghum) form a ternary complex that regulates condensed tannin biosynthesis in sorghum [84,86] (Table 3; Figure 3).

**Table 3:** Candidate genes involved in the biosynthesis and modulation of phenolic compounds and tannins in sorghum seeds during maturation and germination.

Gene/Locus	Type	Function in the Phenylpropanoid/ Flavonoid Pathway	Expected Response During Maturation	Expected Response During Germination	References
*Tan1*	Regulatory (WD40)	Component of the MBW complex; regulates proanthocyanidin biosynthesis	↑↑	↓ or stable	[11,38,84,85,86,90]
*Tan2*	Regulatory (bHLH)	Component of the MBW complex; regulates proanthocyanidin biosynthesis	↑↑	↓ or stable	[11,38,84,85,86,90]
*PAL*	Structural	Entry point of the phenylpropanoid pathway	↑	↑	[11,90,91,92,93]
*C4H*	Structural	Hydroxylation of cinnamic acid	↑	↑	[11,85,90,91,92]
*4CL*	Structural	Carbon flux allocation toward flavonoids and lignin	↑↑	↑	[11,85,90,91,92]
*CHS*	Key structural	Rate-limiting enzyme of flavonoid biosynthesis	↑	↑↑	[11,85,90,91,92,93]
*CHI*	Structural	Conversion of chalcones into flavanones	↑	↑↑	[11,85,90,91,92]
*F3′H*	Structural	Flavonoid diversification	↑	↑↑	[89,90,93]
*DFR*	Structural	Directs metabolic flux toward anthocyanidins and tannins	↑↑	↓	[11,85,90,91,92]
*ANS*	Structural	Conversion of leucoanthocyanidins into anthocyanidins	↑↑	↓	[11,85,90,91,92]
*MYB*	Regulatory	Component of the MBW complex; regulates proanthocyanidin biosynthesis	↑	↑	[11,38,84,85,86,90]
*ABCB28*	Transport/regulatory	Transport and compartmentalization of phenolic compounds	↑	↑	[94]
*LeETR4*	Structural/Receptor	Ethylene receptor involved in hormonal regulation	↑	nr	[89]

*Tan1*: *Tannin1*; *Tan2*: *Tannin2*; *PAL*: phenylanaline amonialyase; *C4H*: cinamato-4-hidroxilase; *4CL*: 4-coumarato-CoA ligase; *CHS*: chalcone synthase; *CHI*: chalcone-flavanone isomerase; *F3′H*: flavanone 3′-hydroxylase; *DFR*: dihydroflavonol-4-reductase; *ANS*: anthocyanidin synthase; *MYB*: it belongs to the family of transcription factors that possess the conserved MYB domain; *ABCB28*: ABC TRANSPORTER B FAMILY MEMBER 28; *LeETR4*: ethylene receptor 4; ↑: genes upregulated; ↓: genes downregulated; ↑↑ genes super expressed. nr: expression not reported.

The production of 3-deoxyanthocyanidins, especially luteolinidin and apigeninidin, is regulated by the *Y1* (*yellow seed 1*) gene located on chromosome 1 [83]. GWAS identified significant QTLs for polyphenol content and antioxidant activity across multiple chromosomes beyond *Tan1* and *Y1*, including loci near homologs of other flavonoid pathway genes on chromosomes 4, 7, and 8 [82,85]. Key structural genes encoding enzymes within the flavonoid pathway have also been identified in sorghum. For example, *CHS* encodes chalcone synthase, which catalyzes the initial step of the flavonoid pathway. The gene *F3′H*, a homolog of maize *Pr1*, encodes flavanone-3′-hydroxylase, an enzyme essential for 3-deoxyanthocyanidin production in maize and also implicated in sorghum biosynthesis of these pigments [86,95]. Expression of these genes is upregulated in pigmented sorghum genotypes [91].

RNA-seq analyses across sorghum cultivars have revealed differential expressions of genes associated with polyphenol biosynthesis and seed coat color, including transcriptional regulators forming co-expression networks linked to tannin and flavonoid content [94]. This network-based approach identifies sets of genes with similar expression patterns, examines correlations between phenotypes and gene modules, infers regulatory networks, and highlights key regulatory genes. RNA sequencing identified 1422 upregulated and 1586 downregulated differentially expressed genes. Several gene modules were correlated with total phenolic content, including 37 differentially expressed genes involved in the phenylpropanoid pathway and 48 in flavonoid biosynthesis. The study further identified central genes such as *ABCB28* (encoding an ABC transporter likely involved in metabolite transport), *PTCD1* (involved in mitochondrial RNA processing and stability), and *ANK* (encoding ankyrin-repeat proteins implicated in multiple plant physiological pathways, including abiotic stress responses) [94].

Transcriptome profiling of sorghum cultivars across grain maturation stages showed that global gene expression varies by developmental stage. Genes involved in phenolic biosynthesis tend to be more highly expressed during early maturation, while the number of downregulated genes increases as maturation progresses. For example, *Tan1* and *Tan2* expressions decreased at the final maturation stage. Notably, although expression of these genes declines toward late maturation, total tannin content increases until the penultimate maturation stage [96].

From a nutritional perspective, combined metabolomic and transcriptomic analyses during sorghum seed development have identified core genes and networks related to starch and protein biosynthesis. Although primarily focused on the endosperm, where starch and protein biosynthesis are most active, these studies establish a basis for understanding genetic coordination of nutrient accumulation [97]. Precise identification of genes and alleles, including *Tan1* and *Tan2* variants, is therefore crucial for breeding, genotype selection, and the development of sorghum lines with targeted tannin and antioxidant profiles for diverse applications, including functional foods and feed [11,85].

### 10.2. Genetic Regulation During Grain Germination

Germination induces substantial changes in the nutritional and functional composition of grains through the coordinated activation of biosynthetic and hydrolytic enzymes, resulting in structural and quantitative modifications of bioactive compounds [58]. During this process, most transcriptional and metabolic activity is localized in the embryo, where the emergence of the radicle and epicotyl occurs and intense gene expression is required. This activity includes the activation of genes involved in secondary metabolism, particularly those associated with phenolic biosynthetic pathways, with the embryo being the main site of flavonoid-related metabolic activity (Figure 3) [98].

The genetic mechanisms underlying changes in phenolic and tannin content during sorghum germination remain relatively underexplored; however, evidence from other cereal species provides relevant mechanistic insights. In cereals, germination activates phenolic biosynthetic pathways and promotes extensive phenolic transformations [60]. For example, significant increases in total phenolics and flavonoids have been reported during germination of buckwheat across different tissues, concomitant with elevated expression of PAL [99]. PAL catalyzes a key entry step of the general phenylpropanoid pathway by converting phenylalanine into cinnamic acid and is therefore essential for flavonoid and phenolic biosynthesis. Increased PAL activity has also been reported during wheat germination [100].

In sorghum, PAL is encoded by an eight-gene family whose expression is regulated by both biotic and abiotic stresses, with higher expression levels associated with stress-resistant genotypes [101]. Analogous to stress responses, PAL activity has been shown to increase during sorghum germination [66]. In addition, genes involved in the phenylpropanoid and flavonoid pathways, including *PAL*, *CHS*, and *F3H*, have exhibited increased expression during germination recovery in sorghum seeds treated with spermidine [93].

Integrated analyses combining transcriptomics, metabolomics, and genome-wide association studies (GWAS) have further identified gene modules associated with phenolic compound accumulation in sorghum. Although these studies primarily focused on hormonal regulation of germination through abscisic acid (ABA) signaling and carbohydrate mobilization, differentially expressed genes were significantly enriched in the phenylpropanoid biosynthesis pathway. At 24 h of germination, phenylpropanoid biosynthesis emerged as one of the most enriched pathways in comparisons between genotypes with contrasting germination capacity, and this enrichment remained significant at 48 h, alongside flavonoid and flavonol biosynthesis pathways (Table 3; Figure 3). Metabolomic profiling further revealed enrichment of flavonoids, organic acids, phenolic acids, and tannins during early germination stages [90].

Given the current knowledge gap regarding gene expression dynamics during sorghum germination, experimental evidence from other cereal species remains valuable for predicting regulatory behaviors of genes involved in phenolic and tannin biosynthesis. Nevertheless, genetic manipulation of sorghum bioactive traits remains complex, as multiple essential genes contribute to the synthesis, regulation, and accumulation of these compounds [86]. The limited availability of sorghum-specific studies on genetic regulation underscores the need for further investigation into molecular mechanisms governing germination-driven bioactive modulation, intending to optimize grain quality, enhancing functional properties, and supporting food security.

## 11. Final Consideration

Germination is a complex physiological process that modulates proximate composition, gene expression, and the synthesis or bioconversion of bioactive compounds in sorghum grains. This process is driven by water uptake, enzyme activation, and the mobilization of nutritional reserves, resulting in significant nutritional and functional changes. Concurrently, germination induces the expression of genes associated with secondary metabolism, particularly those involved in the phenylpropanoid pathway, enhancing the accumulation of bioavailable phenolic compounds.

Key regulatory genes involved in condensed tannin biosynthesis, notably *Tannin1* (*Tan1*) and *Tannin2* (*Tan2*), play a central role in controlling tannin accumulation during grain development. Although primarily associated with seed maturation and pigmentation, germination-driven metabolic reprogramming may modulate the functional activity of these genes, contributing to reduced condensed tannin levels through enzymatic degradation, oxidation, polymerization, and leaching processes.

In parallel, increased activity of biosynthetic enzymes such as phenylalanine ammonia-lyase (PAL), promotes metabolic flux toward phenolic acids and low-molecular-weight flavonoids with higher bioavailability. Collectively, these coordinated molecular and biochemical responses enhance the phenolic profile while reducing antinutritional constraints, thereby improving nutrient bioavailability and supporting the development of sorghum-based functional foods with enhanced health-promoting properties.

## Figures and Tables

**Table 1:** Polyphenolic composition of different sorghum varieties.

Variety	Phenolic Compounds (mg·100 g^−1^)	Individual Phenolic Contents (mg·100 g^−1^)	References
Black Sorghum	TPC: 844.21 TFC: 30.54 TPAC: 257.47 TC: 4.32 Flavanols: 0.239	*Hydrocinnamic acid*: Caffeic acid (2.49), *p*-coumaric acid (2.96), Ferulic acid (1.55); *Hydrobenzoic acids*: Protocatechuic acid (2.55); *3-deoxyanthocyanidin*: Luteolinidin (0.93), Apigeninidin (1.85); *Flavones*: Luteolin (1.23); Apigenin (0.97); *Flavanone*: Naringenin (0.78).	[15]
White Sorghum	TPC: 173.68 TFC: 21.34 TPAC: 96.56 TC: 10.39 Flavanols: 0.172	*Hydrocinnamic acid*: Caffeic acid (1.56), *p*-coumaric acid (0.68), Ferulic acid (1.02); *Hydrobenzoic acids*: Protocatechuic acid (1.63); *3-deoxyanthocyanidin*: Luteolinidin (0.57), Apigeninidin (1.13); *Flavones*: Luteolin (0.93); Apigenin (0.55); *Flavanone*: Naringenin (0.49).	
Pearl White Sorghum	TPC: 191.18 TFC: 15.33 TPAC: 73.84 TC: 12.40 Flavanols: 0.122	*Hydrocinnamic acid*: Caffeic acid (1.43), *p*-coumaric acid (0.86), Ferulic acid (0.81); *Hydrobenzoic acids*: Protocatechuic acid (1.31); *3-deoxyanthocyanidin*: Luteolinidin (0.35), Apigeninidin (0.87); *Flavones*: Luteolin (0.68); Apigenin (0.38); *Flavanone*: Naringenin (0.36).	
Brown Sorghum	TPC: 955.88; TFC: 36.73; TPAC: 835.11; TC: 1.13; Flavanols: 0.245	*Hydrocinnamic acid*: Caffeic acid (3.30), *p*-coumaric acid (1.11), Ferulic acid (2.22); *Hydrobenzoic acids*: Protocatechuic acid (4.63); *3-deoxyanthocyanidin*: Luteolinidin (1.14), Apigeninidin (2.66); *Flavones*: Luteolin (1.56); Apigenin (1.27); *Flavanone*: Naringenin (0.90).	
Red Sorghum	TPC: 1040.73; TFC: 42.84; TPAC: 864.44; TC: 1.44; Flavanols: 0.281	*Hydrocinnamic acid*: Caffeic acid (3.87), *p*-coumaric acid (1.33), Ferulic acid (2.86); *Hydrobenzoic acids*: Protocatechuic acid (5.88); *3-deoxyanthocyanidin*: Luteolinidin (1.28), Apigeninidin (3.74); *Flavones*: Luteolin (1.85); Apigenin (2.24); *Flavanone*: Naringenin (1.86).	
White Sorghum	TPC: 9100 Flavanols: 0.11	*Hydrocinnamic acid*: Chlorogenic acid (0.36), Caffeic acid (0.31), *p*-coumaric acid (0.20); *Hydrobenzoic acids*: Protocatechuic acid (0.32), Gallic acid (0.1); *Flavonols*: quercetin (0.11)	[16]
Red Sorghum	TPC: 170 Flavanols: 26.80	*Hydrocinnamic acid*: Chlorogenic acid (1.05), Caffeic acid (1.0), *p*-coumaric acid (0.68); *Hydrobenzoic acids*: Protocatechuic acid (0.53), Gallic acid (0.2); *3-deoxyanthocyanidin*: Luteolinidin (2.65), Apigeninidin (0.9); *Flavonols*: Quercetin (7.43).	
Brown Sorghum	TPC: 0.02–0.07 TC: 0.49–4.08	*3-deoxyanthocyanidin*: Luteolinidin (0–0.04), Apigeninidin (0–0.03), 5-Methoxyluteolinidin (0–0.07); *Flavones*: 7-Methoxyapigeninidin (0–0.02), Luteolin (0–0.01), Apigenin (0–0.09).	[17]
Orange Sorghum	TPC: 0.01–0.08 TC: 0.25–3.30	*3-deoxyanthocyanidin*: Luteolinidin (0.001–0.02), Apigeninidin (0.002–0.07), 5-Methoxyluteolinidin (0.001–0.02); *Flavones*: 7-Methoxyapigeninidin (0.002–0.02), Luteolin (0.002–0.06), Apigenin (0–0.19).	
Red Sorghum	TPC:0.03–0.05 TC:0.84–2.77	*3-deoxyanthocyanidin*: Luteolinidin (0.005–0.02), Apigeninidin (0.004–0.02), 5-Methoxyluteolinidin (0.005–0.01); *Flavones*: 7-Methoxyapigeninidin (0.002–0.01), Luteolin (0.002–0.09), Apigenin (0–0.0008).	
White Sorghum	TPC: 0.01–0.03 TC: 0.32–1.08	*3-deoxyanthocyanidin*: Luteolinidin (0.001–0.04), Apigeninidin (0.007–0.07), 5-Methoxyluteolinidin (0.006–0.02); *Flavones*: 7-Methoxyapigeninidin (0.005–0.05), Luteolin (0.001–0.03), Apigenin (0.0003–0.06).	
Yellow Sorghum	TPC: 0.03–0.09 TC: 0.99–3.65	*3-deoxyanthocyanidin*: Luteolinidin (0.001–0.04), Apigeninidin (0.003–0.03), 5-Methoxyluteolinidin (0–0.05); *Flavones*: 7-Methoxyapigeninidin (0.002–0.02), Luteolin (0.001–0.02), Apigenin (0–0.04).	
Red Sorghum	TPC: 923	*Hydrocinnamic acid*: Caffeic acid (232), *p*-coumaric acid (178), Ferulic acid (62); Chlorogenic acid (32), Feruloylquinic acid (52); *Flavanone*: Naringenin-7-*O*-glucosid (367).	[18]
Brown Sorghum	TPC: 329	*Hydrocinnamic acid*: Caffeic acid (105), *p*-coumaric acid (52), Ferulic acid (67); Chlorogenic acid (20), Feruloylquinic acid (72); *Flavanone*: Naringenin-7-*O*-glucosid (13).	
White Sorghum	TPC: 425	*Hydrocinnamic acid*: Caffeic acid (111), *p*-coumaric acid (91), Ferulic acid (61), Chlorogenic acid (75), Feruloylquinic acid (76); *Flavanone*: Naringenin-7-*O*-glucosid (11).	
Red Sorghum	ND	*Hydrocinnamic acid*: Caffeic acid (5.15), *p*-coumaric acid (7.10), Ferulic acid (9.15), Chlorogenic (1.15); *Hydrobenzoic acids*: 4-hydroxybenzoic (1.9), Protocatechuic acid (8.35), Gallic acid (5.90), Sinapic acid (1.05), Syringic acid (0.55), Vanillic acid (2.3); *Flavones*: Luteolin (0.13), Apigenin (0.054), Catechin (0.36), Kaempferol (0.033); *Flavanone*: Naringenin (0.058), Vitexin (0.050); *Flavonols*: Quercertin (0.017), Rutin (0.042).	[19]
White Sorghum		*Hydrocinnamic acid*: Caffeic acid (2.15), *p*-coumaric acid (14.9), Ferulic acid (29.3), Chlorogenic (2.5), *t*-cinnamic acid (1.15); *Hydrobenzoic acids*: 4-hydroxybenzoic (1.15), Protocatechuic acid (14.25), Gallic acid (1.65), Sinapic acid (1.75), Syringic acid (2.50); *Flavones*: Luteolin (0.39), Catechin (0.46), Kaempferol (0.043); *Flavanone*: Naringenin (0.11), Vitexin (0.090); *Flavonols*: Quercertin (0.049), Rutin (0.16).	

TPC: total phenolic content in GAE (gallic acid equivalents); TFC: total flavonoid content in RE (rutin equivalents); TPAC: total proanthocyanidin content in CE (catechin equivalents); TC: tannin content; ND—not determined.

**Table 2:** Effect of germination on the polyphenolic composition of different sorghum varieties.

Sorghum Variety	Germination Conditions	Effect on Phenolic Compounds	Effect on Individual Phenolic Content (µg·g^−1^)	References
Red Sorghum	Soaking (1:10) grain/water at 30 °C for 12 h. Germinated at 30 °C for 12, 24, 36 and 48 h.	TPC: ↑ 48.84% Flavonoid: ↑ 43.88% Anthocyanin: ↑ 49.04%	*Free phenolic compounds*—*Phenolic acids*: Gallic acid (↑ from 0 to 37.87), Chlorogenic acid (↑ from 99.98 to 118.60), Caffeic acid (↑ from 34.55 to 199.96), Protocatechuic acid (↑ from 27.96 to 69.99), *p*-coumaric acid (↑ from 5.67 to 15.09), *p*-hydroxybenzoic acid (↑ from 6.72 to 8.21), Vanillic acid (↑ from 0 to 8.66), Syringic acid (↑ from 64.44 to 64.80), Ferulic acid (↑ from 38.28 to 52.0), Salicylic acid (↑ from 0 to 32.18), *O*-coumaric acid (↓ from 12.95 to 12.32); *Flavonoids*: Daidzin (↓ from 54.48 to 39.53); *Flavones*: Luteolin (↓ from 8.47 to 4.82), Apigenin (↑ from 7.79 to 9.92); *Flavanones*: Naringenin (↑ from 70.08 to 76.66); *Flavonols*: Quercetin (↓ from 80.26 to 79.49), Rutin (↑ from 17.18 to 36.57), Kaempferol (↑ from 15.46 to 15.50), Myricetin (↑ from 11.27 to 25.37), *Anthocyanins*: Cyanidinchloride (↑ from 2.49 to 5.59), Cyanidin-3-rutinoside (↑ from 45.90 to 61.53), Petunidin-3-glucosidechloride (↑ from 11.50 to 15.73), peonidin-3-glucosidechloride (↑ from 22.51 to 27.25).	[62]
Hulled Sorghum	Soaked (1:3) grain/water for 16 h at 27 °C. Germinated for 48 h or 72 h at 27 °C and 90% relative humidity.	TPC: ↑ 40,10%	*Free phenolic compounds*—*Phenolic acids*: 1-3-*O*-dicaffeoylglycerol (↓ from 270.1 to 23.83), Caffeic acid (↓ from 35.92 to 3.90); *Flavan-3-ols*: Catechin (↑ from 0 to 33.99); *Flavonols*: Taxifolin(↑ from 1327.69 to 2233.5); *Flavones*: Apingenin: (↑ from 23.86 to 29.79), Hispidulin isomer 1 (↑ from 58.23 to 179.52), Hispidulin isomer 2 (↑ from 188.27 to 281.74), Luteolin (↑ from 373.47 to 435.66); *Proanthocyanidins*: Procyanidin dimer (↑ from 0 to 26.96); *Flavanones*: Eriodictyol (↑ from 40.51 to 219.26), Naringenin (↑ from 5.83 to 37.82), Kaempferol (↑ from 4.48 to 18.71); *Flavanonols*: Dihydromyricetin 3-*O*-Rhamnoside (↑ from 31.98 to 41.13); *3-deoxyanthocyanidins*: Luteolinidin (↑ from 2.23 to 7.64), Apigeninidin (↓ from 6.51 to 1.29), 5-methoxy-luteolinidin (↓ from 6.02 to 3.68), 7-methoxy-apigeninidin (↑ from 1.58 to 20.18).	[66]
		TPC: ↑ 200.5%	*Bound phenolic compounds*—*Flavonols*: Taxifolin (↑ from 0 to 13.02); *Flavones*: Hispidulin isomer 1 (↑ from 0 to 62.32), Hispidulin isomer 2 (↑ from 7.49 to 34.65), Luteolin (↑ from 67.65 to 103.23); *Flavanones*: Eriodictyol (↑ from 0 to 21.64), Naringenin (↑ from 11.47 to 49.70).	
Red Sorghum	Soaked (1:2.5) grain/water for 24 h/21 °C. Germinated for 48 h or 24 h at 30 °C.	TPC: ↓ 75.72%	*Phenolic acids*: Protocatechuic acid (↑ from 0 to 350), Caffeic acid (↓ 290 to 47); *Flavones*: Apigenin-*C*-pentosyl-*C*-hexoside (↓ from 3225 to 698); *Flavanones*: Naringenin-*O*-hexoside (↓ 1686 to 24).	[67]
White Sorghum	Germination: 24/36 or 48 h/23 °C	TPC: ↑ 171,43% TFC: ↑ 11,11%	*Phenolic acids*: Caffeic acid (↓ from 0.09 to 0), Chlorogenic acid (↑ from 0.03 to 0.05), Gallic acid (↑ from 0 to 0.05), *p*-coumaric acid (↑ from 0.15 to 0.23); *Flavan-3-ols*: Epicatechin (↓ from 0.85 to 0.16); *Flavanones*: Naringenin (↑ from 0.12 to 0.19); *Flavonoids*: Daidzein (↓ from 0.03 to 0), Genistein (↑ from 0.15 to 0.23); *Flavonols*: Quercetin (↓ 0.06 to 0.02), Rutin (↑ from 0 to 0.06).	[68]
Red Sorghum	Soaked (1:2.5) 20 h/(23 °C) Germination: 72 h/23 °C	Total Flavones: ↓ 87.24% Total Flavanones: ↓ 73.36% 3-deoxyanthocyanidins: ↓ 30.04%	*Flavanones*: Eriodyctiol (↓ from 0.242 to 0.082), Naringenin (↓ from 0.170 to 0.022); *Flavones*: Luteolin (↓ from 0.064 to 0.007), Apigenin (↓ from 0.034 to 0.004); *3-deoxyanthocyanidins*: Luteolinidin (↓ from 0.022 to 0.012), Apigeninidin (↓ from 0.018 to 0.013), 5-methoxy-luteolinidin (↓ from 0.024 to 0.014), 7-methoxy-apigeninidin (↓ from 0.018 to 0.016).	[69]
Kafir (red pericarp)	Soaked: - Germination: 0 h, 72 h and 144 h/16 °C	TPC: ↑ 47.94% Flavonoid: ↑ 4.54%	*^£^Flavonoids:* Hesperidin/neohesperidin: ↓ 5 × 10^4^ to 2.39 × 10^4^, Naringin glucoside 1: ↓ 2.12 × 10^4^ to 5.27 × 10^3^, Naringenin 4′-O-glucuronide: ↓ 3.14 × 10^5^ to 4.66 × 10^4^, Daidzin: ↓ 3.31 × 10^6^ to 1.65 × 10^6^, Apigenin: ↓ 1.52 × 10^5^ to 6.18 × 10^4^ *Phenolic acids:* Caffeic acid glucoside: ↑ 7.33 × 10^3^ to 9.53 × 10^3^, 4-Hydroxybenzoic acid: ↓ 1.71 × 10^5^ to 6.63 × 10^4^, Caffeoylquinic acid isomer: ↓ 2.56 × 10^4^ to 1.39 × 10^4^, 4 Hydroxybenzaldehyde: ↑ 7.01 × 10^5^ to 2.33 × 10^6^, Caffeic acid: ↓ 5.67 × 10^5^ to 3.52 × 10^5^, Dihydroferulic acid 4-O-glucuronide: ↓ 6.22 × 10^4^ to 1.23 × 10^4^, *p*-Coumaric acid: ↓ 3.05 × 10^5^ to 2.88 × 10^5^, Isoferulic acid: ↑ 4.53 × 10^5^ to 5.50 × 10^5^	[70]
Caudatum (white pericarp)		Flavonoid: ↑ 1.21%	*^£^Flavonoids:* Hesperidin/neohesperidin: ↓ 4.75 × 10^4^ to 3.91 × 10^4^, Naringin glucoside 1: ↑ 5.82 × 10^3^ to 7.49 × 10^3^, Naringenin 4′-O-glucuronide: ↑ 1.09 × 10^4^ to 1.88 × 10^4^, Daidzin: ↓ 2.52 × 10^6^ to 9.68 × 10^5^, Apigenin: ↓ 8.25 × 10^4^ to 5.15 × 10^4^ *Phenolic acids*: Caffeic acid glucoside: ↑ 1.04 × 10^4^ to 1.94 × 10^4^, 4-Hydroxybenzoic acid: ↓ 1.79 × 10^5^ to 7.96 × 10^4^, Caffeoylquinic acid isomer: ↓ 6.80 × 10^3^ to 2.54 × 10^4^, Hydroxybenzaldehyde: ↑ 1.34 × 10^6^ to 5.20 × 10^6^, Caffeic acid: ↓ 5.29 × 10^5^ to 2.53 × 10^5^, Dihydroferulic acid 4-O-glucuronide: ↓ 6.11 × 10^4^ to 2.39 × 10^4^, *p*-Coumaric acid: ↑ 2.72 × 10^5^ to 2.76 × 10^5^, Isoferulic acid: ↓ 5.06 × 10^5^ to 2.38 × 10^4^	
Kafir-caudatum (white pericarp)		Flavonoid: ↑ 0.61%	*^£^Flavonoids:* Hesperidin/neohesperidin: ↑ 3.41 × 10^4^ to 9.0 × 10^3^, Naringin glucoside 1: ↑ 1.29 × 10^4^ to 1.33 × 10^4^, Naringenin 4′-O-glucuronide: ↓ 1.07 × 10^4^ to 6.87 × 10^3^, Daidzin: ↓ 3.45 × 10^6^ to 1.74 × 10^6^, Apigenin: ↓ 2.33 × 10^6^ to 4.66 × 10^5^ *Phenolic acids:* Caffeic acid glucoside: ↓ 3.37 × 10^4^ to 2.12 × 10^4^, 4-Hydroxybenzoic acid: ↑ 4.70 × 10^4^ to 7.06 × 10^4^,↓ 6.80 × 10^3^ to 2.54 × 10^4^, Hydroxybenzaldehyde: ↑ 1.68 × 10^5^ to 3.48 × 10^6^, Caffeic acid: ↓ 7.11 × 10^5^ to 4.27 × 10^5^, Dihydroferulic acid 4-O-glucuronide: ↓ 8.69 × 10^4^ to 3.39 × 10^4^, *p*-Coumaric acid: ↑ 4.36 × 10^5^ to 4.67 × 10^5^, Isoferulic acid: ↑ 1.07 × 10^6^ to 1.18 × 10^6^	

TPC: total phenolic content in GAE (Gallic acid equivalents); TFC: total flavanoid content in RE (Rutin equivalents); ↑: increase; ↓: decrease; ^*£*^ data expressed as total relative ion abundance.

## Data Availability

No new data were created or analyzed in this study. Data sharing is not applicable to this article.
